# Increasing Number and Proportion of Adverse Obstetrical Outcomes among Women Living with HIV in the Ottawa Area: A 20-Year Clinical Case Series

**DOI:** 10.1155/2016/1546365

**Published:** 2016-07-27

**Authors:** Sarah Buchan, Katherine A. Muldoon, Johanna N. Spaans, Louise Balfour, Lindy Samson, Mark Walker, D. William Cameron

**Affiliations:** ^1^Clinical Epidemiology Program, Ottawa Hospital Research Institute, The Ottawa Hospital, Ottawa, ON, Canada K1H 8L6; ^2^Department of Medicine, Division of Infectious Disease, The Ottawa Hospital, Ottawa, ON, Canada K1H 8L6; ^3^School of Epidemiology, Public Health and Presentation Medicine, University of Ottawa, Ottawa, ON, Canada K1H 8L6; ^4^Department of Obstetrics and Gynaecology, The Ottawa Hospital, Ottawa, ON, Canada K1H 8L6; ^5^Department of Pediatrics, Division of Infectious Diseases, Children's Hospital of Eastern Ontario, University of Ottawa, Ottawa, ON, Canada K1H 8L1

## Abstract

*Background*. The prevalence and associated risks with adverse obstetrical outcomes among women living with HIV are not well measured. The objective of this study was to longitudinally investigate the prevalence and correlates of adverse obstetrical outcomes among women with HIV.* Methods*. This 20-year (1990–2010) clinical case series assessed the prevalence of adverse obstetrical outcomes among pregnant women with HIV receiving care at The Ottawa Hospital (TOH). General estimating equation modeling was used to identify factors independently associated with adverse obstetrical outcomes, while controlling for year of childbirth clustering.* Results*. At TOH, there were 127 deliveries among 94 women (1990–2010): 22 preterm births, 9 births with low birth weight, 12 births small for gestational age, and 4 stillbirths. Per year, the odds of adverse obstetrical outcomes increased by 15% (OR: 1.15, 95% CI: 1.03–1.30). Psychiatric illness (AOR: 2.64, 95% CI: 1.12–6.24), teen pregnancy (AOR: 3.35, 95% CI: 1.04–1.46), and recent immigrant status (AOR: 7.24, 95% CI: 1.30–40.28) were the strongest correlates of adverse obstetrical outcomes.* Conclusions*. The increasing number and proportion of adverse obstetrical outcomes among pregnant women with HIV over the past 20 years highlight the need for social supports and maternal and child health interventions, especially among adolescents, new immigrants, and those with a history of mental illness.

## 1. Introduction

High rates of adverse obstetrical outcomes have been reported among women with human immunodeficiency virus (HIV) [1–3]. The introduction of antiretroviral therapy (ART) has improved the health of mothers and reduced the risk of perinatal transmission. However, despite these clinically significant advancements, women living with HIV continue to be at heightened risk for infant morbidity and mortality compared to the general population [4, 5]. Some studies have begun to explore the role of highly active antiretroviral therapy and protease inhibitor- (PI-) based ART regimens as a risk factor for low birth weight and premature delivery [2, 6, 7]; however, the specific association between ART and adverse obstetrical outcomes remains unclear but is likely attributed to multiple pathways. In high-income settings, common attributable risk factors include maternal comorbidities, hazardous substance use, and psychosocial stressors, all of which are more common in women with HIV [8].

The Public Health Agency of Canada reported that, between 1996 and 2009, there has been an average per-year increase of 3% (ages 18 to 35) and 11% (ages 36–49) in HIV prevalence among women of childbearing age in Canada [9]. For the Canadian healthcare system to meet the evolving needs of women living with HIV, a comprehensive examination of access to sexual and reproductive healthcare is needed. Important screening opportunities have happened through antenatal screening and immigration, both considered care points to engage women in HIV testing, treatment, and care [10].

In Eastern Ontario, the regional adult HIV healthcare facility is housed at the General Campus of The Ottawa Hospital (TOH). Since 1988, TOH has provided centralised regional, comprehensive, multidisciplinary healthcare, medical treatment, and subspecialty referrals for people living with HIV. These include services for maternal, antenatal, and neonatal healthcare before infant referral to the Children's Hospital of Eastern Ontario (CHEO). Since 2010, medical HIV healthcare has gradually migrated to the primary care community setting, with some changes in provision of multidisciplinary supports and subspecialty referrals.

The purpose of this study was to (1) clinically and demographically characterize all women living with HIV who accessed obstetrical care at TOH over a 20-year period (1990–2010) and (2) assess the prevalence and correlates of adverse obstetrical outcomes among women living with HIV.

## 2. Methods

### 2.1. Study Design and Data Collection

This study was a retrospective clinical case series conducted at the Immunodeficiency HIV/AIDS Clinic at The Ottawa Hospital (TOH), with a multidisciplinary healthcare team providing care and services to people living with HIV. The charts of all women with HIV seen at the clinic were reviewed and any women with a recorded pregnancy on file were included. Data was collected in June 2011 and covered a twenty-year period between June 1990 and July 2010. Data were abstracted from patient charts and electronic medical records using a standardized case report form. Data from the completed forms were entered into Excel*™* spreadsheet by two research assistants and checked by the research team members for completeness and accuracy. The analytical sample was restricted to pregnancies that advanced to delivery (>20 weeks). Each additional birth (e.g., subsequent delivery) is included as a new individual record but analyzed as a dependent event. The unit of analysis is each individual delivery.

### 2.2. Variable Selection

#### 2.2.1. Dependent Variable

Adverse obstetrical outcomes among pregnancies that advanced to delivery included preterm birth (<37 weeks of gestation), low birth weight (<2500 g), being small for gestational age (<10th percentile) [11], and stillbirth. A composite outcome was derived to compare healthy deliveries to those with at least one of the four types of adverse obstetrical outcomes. In addition to counting particular outcomes over time, a composite outcome of these four was used to increase statistical power to detect trends and associations.

#### 2.2.2. Independent Variables

A continuous variable was computed to assess gravidity defined as the number of times that a woman has been pregnant (including abortions and miscarriages), and parity was defined as the number of pregnancies past 20 weeks regardless of outcome. Both gravidity and parity were measured at the time of birth. Women who experienced a subsequent obstetrical event (e.g., another delivery, miscarriage, or abortion) had their scores adjusted for each additional event.

Mode of delivery compared those who delivered vaginally to those who underwent a caesarean section (elective or emergent). Maternal age was categorized into a 3-level variable to measure teen pregnancy (<20 years), low-risk pregnancy (20–35 years), and high-risk pregnancy (≥36 years), consistent with standard Canadian prenatal guidelines. A continuous variable recorded year of delivery.

A 3-level variable was derived to measure immigration status comparing nonimmigrants, recent immigrants (<10 years), and long-term immigrants (≥10 years). A binary variable was used to identify African migrants (yes versus no) to account for the large proportion of African diaspora coming from countries with generalized HIV epidemics. A housing variable was derived to compare those who were stably housed compared to those who were unstably housed (i.e., homeless, living in a shelter or in subsidized housing). Additional variables recorded whether the patient had active provincial health insurance (yes versus no), whether or not they were living with a partner (yes versus no), and whether they had a history of intravenous drug use (yes versus no) or smoking (yes versus no).

HIV diagnosis was dichotomized to measure if the woman had been diagnosed before the pregnancy or during pregnancy/after delivery. CD4 cell count was categorized into <200 *μ*L, 200–499 *μ*L, and >500 *μ*L and virological suppression was defined as having <50 copies/*μ*L. Drug regimens were recorded as any ART (yes versus no), any ART combination with nucleoside reverse-transcriptase inhibitors (NRTI) (yes versus no), NRTIs only (yes versus no), nonnucleoside reverse-transcriptase inhibitors (NNRTI-based) (yes versus no), and protease inhibitors (PI-based) (yes versus no).

A series of binary variables were used to measure key clinical comorbidities at the time of birth including hepatitis A, hepatitis B, hepatitis C, psychiatric history (both preexisting illness preconception and postpartum illness following childbirth) of mood or anxiety disorders as consistent with the Diagnostic and Statistical Manual of Mental Disorders [12], history of sexually transmitted infections (gonorrhea, chlamydia, herpes, human papilloma virus, etc.), history of TB diagnosis (latent and active), history of malaria, diabetes (type I, type II, or gestational), and hypertension.

### 2.3. Data Analysis

Statistical analyses were performed using SAS (version 9.3). Frequencies and proportions were used to measure all categorical variables. Means and ranges were used to display variation in continuous variables. Nonnormality among continuous variables was assessed using the Hosmer-Lemeshow Goodness-of-Fit test.

All missing data are recorded in the descriptive tables (*m* = missing); however, only those with complete data for the dependent and independent variables are included in the logistic regression modeling. Bivariable and multivariable general estimating equation (GEE) modeling was used to account for the within-year correlation structure of childbirth clustering. Under GEE, binomial distribution and logit transformation were utilized. Bivariable logistic regressions were run to investigate the strength of association between each of the independent variables and the composite measure of adverse obstetrical outcomes while controlling for year of birth. Odds ratios (OR) and 95% confidence intervals (CI) are used to display the precision of the estimates. Multivariable models included covariates considered to be significant (*p* > 0.05) at the bivariable level using adjusted odds ratios (AOR) and 95% CI.

### 2.4. Ethics

This study received research ethics board approval from The Ottawa Hospital Research Ethics Board (OHREB) prior to data abstraction (OHREB #2010527-01H). The OHREB waived the need for written patient consent. Any patient identifiers were not used and all data were anonymized. Each patient record was assigned an independent study number and only month and year of birth were extracted.

## 3. Results

### 3.1. Descriptive Statistics

From 1990 to 2010, 94 women had a total of 145 pregnancies: 127 advanced to delivery and 18 terminated within the first trimester (11 abortions and 7 miscarriages). Among the 127 pregnancies that advanced to delivery, 22 (17.74%) were preterm, 9 (7.09%) were of low birth weight, 12 (9.45%) were small for gestational age, and 4 (3.15%) were stillbirth. A total of 27 (21.26%) individual births resulted in at least one negative outcome as measured by the composite outcome (Table 1).

**Figure 1:**
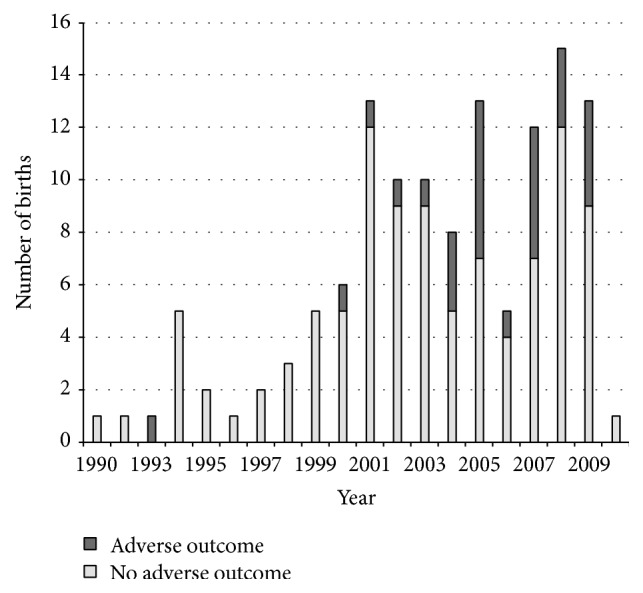
Distribution of obstetrical outcomes by year of childbirth.

Over time, there was an increase in both the number of births and the prevalence of adverse obstetrical outcomes. In the first decade (1990–1999), there was a cumulative total of 21 births, 4.76% of which had adverse obstetrical outcomes. In the second decade (2000–2010), there was a cumulative total of 106 births, 24.52% of which had adverse obstetrical outcomes. For each additional year, the odds of adverse obstetrical outcomes increased by 15% (OR: 1.15, 95% CI: 1.03–1.30).  Figure 1 displays a graph of the distribution of adverse obstetrical outcomes by year.

Table 2 displays the descriptive results among the 127 pregnancies that advanced to delivery. For 86 (65.35%) women, it was their first pregnancy, and for 83 (67.72%) it was their first term birth (parity).

Half of the deliveries (43.41%) were vaginal and 48.03% were by caesarean section (elective and emergent). There were 5 (3.93%) teen pregnancies, 96 (75.60%) pregnancies among women aged 20–35 years, and 26 (20.47%) pregnancies among women aged 36 years and older. There were 82 (56.55%) recent immigrants; 69.29% of the entire sample were African migrants with the most common countries of origin being the Democratic Republic of Congo, Ethiopia, and Somalia. Most women (53.54%) were living with a partner, and 103 (83.06%) were considered stably housed. The majority of women (65.35%) were diagnosed with HIV preconception. As a measure of immunology, 47 (37.01%) had CD4 counts >500 *μ*L, and, among the 107 for whom we had data on HIV viral load, 72 (56.69%) were determined to have achieved virological suppression. A total of 101 (79.53%) women were on ART at the time of birth, 96 (75.59%) on any combination with NRTIs, 14 (11.02%) on NRTIs only, 12 (9.45%) on a NNRTI-based regimen, and 81 (63.78%) on PI-based regimen. The majority (78.74%) of women gave birth during the era of highly effective ART (>2001).

The most prevalent comorbidities were a history of TB infection (21.26%), followed by a history of malaria (17.32%), and a psychiatric history (16.54%).

### 3.2. Bivariable and Multivariable Analyses

Table 3 displays the bivariable and multivariable GEE analyses identifying factors most strongly associated with adverse obstetrical outcomes. Using GEE modeling to control for clustering by year of birth, teen pregnancy (OR: 2.26, 95% CI: 1.00–5.09), recent immigrant status (OR: 5.88 95% CI: 1.26–27.36), and psychiatric history (OR: 2.77, 95% CI: 1.22–6.33) all increased the odds of adverse obstetrical outcomes. In the multivariable analyses, the factors most strongly and significantly associated with adverse obstetrical outcomes were teen pregnancy (AOR: 3.35, 95% CI: 1.04–1.46), recent immigrant status (AOR: 7.24, 95% CI: 1.30–40.28), and psychiatric history (AOR: 2.85, 95% CI: 1.23–6.61).

## 4. Discussion

This study documented an increasing number of births to women living with HIV. While the Canadian healthcare system provides coverage for HIV medical care and antenatal services, this study also found that over 20% of pregnant women living with HIV experienced at least one adverse obstetrical outcome between 1990 and 2010, with the number of adverse obstetrical events increasing over time by as much as 15%. Over 50% of the sample were recent immigrants to Canada, a factor that was associated with over 7 times the odds of adverse obstetrical outcomes. This is a population that can benefit from screening at the time of immigration and linkages to healthcare. Additional risk factors included teen pregnancy and mental illness.

The high prevalence of adverse obstetrical outcomes among this population is concerning. Data from the Ottawa Public Health Unit birth registry show that adverse obstetrical outcomes among the general obstetrical population are significantly lower. For example, the prevalence of premature birth, low birth weight, being small for gestational age, and stillbirth is 9.40%, 6.50%, 7.40%, and 0.54%, respectively, compared to our sample of women living with HIV and the higher prevalence of premature birth (17.32%), low birth weight (7.09%), being small for gestational age (9.45%), and stillbirth (3.15%) [10].

In the era of highly active antiretroviral therapy, more women with HIV are choosing to have children and are in need of safe and supportive services for reproductive health [13]. Despite advancements in antiretroviral therapy, many pregnant women continue to feel stigmatized and judged by healthcare providers. With optimal support from healthcare providers, women living with HIV can proactively plan for pregnancies that minimize risk to themselves and their infants [14]. The Canadian HIV Pregnancy Planning Guidelines [15] were developed to support healthy pregnancies for women living with HIV with a focus on preconception health, prevention of perinatal transmission to infants, sexual transmission to partners, and infertility. The results from this study document the high burden of adverse obstetrical outcomes among pregnant women living with HIV and support the expansion of these guidelines to include antenatal medical healthcare and social supports to reduce the risk of adverse obstetrical outcomes.

It has been estimated that almost 15% of new and prevalent HIV infections happen among immigrant populations within Canada [9]. The majority of this sample (56.55%) had immigrated to Canada in the last 10 years and were identified as a group with significantly higher odds of obstetrical complications. The dual stigma of HIV infection and recent immigration has been shown to decrease uptake of social and health services [9]. HIV testing and screening programs are routinely conducted for immigration and prenatal purposes in Canada [16], with Ontario now testing over 97% of immigrant women who are pregnant. Immigration can be an effective point of contact where women living with HIV interface with the healthcare system and can be connected to sexual and reproductive healthcare services that include antenatal and obstetrical care.

Historically, women with HIV have higher documented prevalence of additional comorbidities, including hepatitis B and hepatitis C, sexually transmitted infections, and mental illness. Mental health issues have also proven to be important factors that influence adherence to ART and contribute to underutilizing antenatal care and are often markers for other comorbidities [17]. Additionally, the prevalence of mental illness is significantly associated with preterm birth and infants small for gestational age [18, 19]. Over 16% of this study population had a history of psychiatric illness, a factor that was independently associated with increased odds of adverse obstetrical outcomes. In this study, the case definition for psychiatric illness included women with preexisting condition preconception and postpartum condition following childbirth. We were unable to disaggregate the data. This study has demonstrated that both the prevalence of psychiatric illness and the adverse obstetrical outcomes are high among women living with HIV, for which development and support of specialized programs are warranted.

Adolescent pregnancies are known to have higher rates of obstetrical complications including preterm labour, anemia, hypertension, other disorders during pregnancy, and low infant birth weight [20]. Additionally, social factors such as early sexual debut [8], limited control over sexual decision-making, and the social stigmatization of teen pregnancy [21] are all known to decrease access to care and heighten risk for the youth. All of these features are likely magnified among female youth living with HIV. In our study, there were five births among teenagers who experienced significantly higher odds of adverse obstetrical outcomes. Even though the youth represent a smaller proportion of pregnancies, they are a group identified to be at increased risk of harm and in need of services that reduce barriers to sexual and reproductive healthcare [22].

In our study, almost 80% of women were on ART at the time of delivery. There is an ongoing debate regarding PI-based ART regimes and their role in obstetrical outcomes particularly linked with fetal growth [6, 23]. In this sample, 63.78% of women were taking PIs and there was a marginally significant increase in the frequency of adverse obstetrical outcomes; however, this was not significant in the regression modeling.

## 5. Limitations

There are several limitations that must be acknowledged. TOH is the only regional referral hospital for women with HIV and other high-risk pregnancies. However, clinical case series are observational studies without a comparison population making them prone to bias, particularly selection bias. This limitation is also reflected in the fact that this case series has a relatively small sample size (*n* = 127 births) which limits statistical power to detect differences between women who did and did not experience an adverse obstetrical outcome. Additionally, the primary dependent variable was a cumulative measure of any adverse obstetrical outcome over a 20-year period. The fact that most pregnancies occurred after the year 2000 and that most adverse outcomes were situated in that group is of interest. There are other adverse obstetrical outcomes that we did not track, such as anemia, third-trimester bleeding, maternal blood loss, or neonate APGAR score at one and five minutes. This case series also does not include information on perinatal HIV transmission. All women with HIV from the clinic were included; however, it is possible that pregnancies that advanced to delivery may not have been recorded in the charts or the women may have delivered their baby outside of TOH facilities. As the outcomes of the potential missing pregnancies are unknown, we are unable to conclude whether the results would overestimate or underestimate the proportion of an adverse obstetrical outcome. Exposure to ART is a complex issue that is difficult to examine. In-depth investigations into time of diagnosis, time of starting ART, each drug combination, and length of exposure to the drug are very interesting and relevant areas for future investigation. Finally, administrative data are prone to missingness, but in place of deleting missing cases they were included in the descriptive statistics and only excluded during bivariable and multivariable analyses.

## 6. Conclusions

While Canada has low prevalence of adverse obstetrical outcomes, key marginalized groups, including pregnant women living with HIV, remain a population with a high burden of obstetrical complications [22]. With the development and provision of effective antiretroviral therapy (ART), with widespread access to simple, safe, and effective ART through primary healthcare in the community setting, more women with HIV are able to plan for healthy pregnancies that reduce the risk of mother-to-child transmission. However, this study indicates that despite the clinical success of preventing mother-to-child transmission and effective HIV healthcare, there remains a high burden of adverse obstetrical outcomes. It is necessary to ensure that women living with HIV are able to access antenatal care and social supports to improve the chances of a healthy pregnancy and birth. This study suggests that, among women with HIV, preterm births among other adverse obstetrical outcomes are increasing in both number and proportion over time. These adverse outcomes may be averted through comprehensive reproductive and antenatal healthcare, and specialized programming for recent immigrants, those with mental illness, and adolescents may improve outcomes. Identifying and supporting families affected by HIV may avert subsequent costs and offer significant individual and societal benefits in maternal-child health.

## Figures and Tables

**Table 1:** Obstetrical outcomes among 127 births from women living with HIV from 1990 to 2010.

Variables	*n* (%)
Total deliveries	
Healthy	100 (78.74%)
Adverse obstetrical outcome^1^	27 (21.26%)
Preterm (<37 weeks)	22 (17.32%)
Low birth weight (<2500 g)	9 (7.09%)
Small for gestational age (<10th percentile)	12 (9.45%)
Stillbirth	4 (3.15%)

^1^Adverse obstetrical outcome is a composite outcome and events are not mutually exclusive. Participants with ≥1 event are counted as “yes.”.

**Table 2:** Descriptive statistics associated with adverse obstetrical outcomes among women living with HIV (*n* = 127 deliveries)

Variables (*m* = missing)^1^	*n* (%)^2^	Yes (*n* = 27)	No (*n* = 100)	*p* value
*Obstetrical factors at time of birth*				
Parity				
1	86 (67.72)	19 (70.37)	67 (67.00)	0.812
2	29 (22.83)	5 (18.52)	24 (24.00)	
3+	12 (9.45)	3 (11.11)	9 (9.00)	
Gravidity				
1	83 (65.35)	16 (59.26)	67 (67.00)	0.755
2	32 (25.20)	8 (29.63)	24 (24.00)	
3+	12 (9.45)	3 (11.11)	9 (9.00)	
Mode of delivery (*m* = 11)				
Vaginal	55 (47.41%)	12 (44.44)	43 (48.31)	0.724
Caesarean section	61 (52.59%)	15 (55.56)	46 (51.69)	
*Sociodemographic variables*				
Age at delivery				
<20 years	5 (3.93%)	2 (7.41)	3 (3.00)	0.484
20–35 years	96 (7.56%)	19 (70.37)	77 (77.00)	
36+ years	26 (20.47%)	6 (22.22)	20 (20.00)	
Ethnicity				
African migrant	88 (69.29%)	19 (70.37)	69 (69.00)	0.891
Other	39 (30.71%)	8 (29.63)	31 (31.00)	
Immigrant status				
Canadian born	46 (31.72)	3 (11.11)	40 (40.00)	0.004
<10 years	82 (56.55)	22 (81.48)	47 (47.00)	
≥10 years	17 (11.72)	2 (7.41)	13 (13.00)	
Health insurance				
Yes	123 (96.85%)	26 (96.30)	97 (97.00)	1.00
No	4 (3.15%)	1 (3.70)	3 (3.00)	
Living with partner (*m* = 27)				
Yes	68 (68.00%)	14 (66.67)	54 (68.35)	0.883
No	32 (32.00%)	7 (33.33)	25 (31.65)	
Housing (*m* = 11)				
Stable	103 (88.79%)	20 (86.96)	83 (89.25)	0.720
Unstable	13 (11.21%)	3 (13.04)	10 (10.75)	
Intravenous drug use				
Yes	7 (5.51)	2 (7.41)	5 (5.00)	0.640
No	120 (94.49)	25 (92.59)	95 (95.00)	
History of smoking				
Yes	13 (10.24)	5 (18.52)	8 (8.00)	0.148
No	114 (89.76)	22 (81.48)	92 (92.00)	
*HIV related variables*				
HIV diagnosis preconception (*m* = 25)				
Yes	83 (81.37%)	18 (78.26)	65 (82.28)	0.762
No	19 (18.63%)	5 (21.74)	14 (17.72)	
CD4 cell count (*m* = 21)				
<200	12 (11.32%)	1 (3.85)	11 (13.75)	0.428
200–499	47 (44.34%)	12 (46.15)	35 (43.75)	
>500	47 (44.34%)	13 (50.00)	34 (42.50)	
Virological suppression (*m* = 20)				
Yes	72 (67.29%)	20 (76.92)	52 (64.20)	0.229
No	35 (32.71%)	6 (23.08)	29 (35.80)	
ART regimen				
Any ART (yes versus no)	101 (79.53)	22 (81.48)	79 (79.00)	0.777
NRTI (yes versus no)	96 (75.59)	20 (74.07)	76 (76.00)	0.836
NRTI only (yes versus no)	14 (11.02)	1 (3.70)	13 (13.00)	0.171
NNRTI-based (yes versus no)	12 (9.45)	0 (0)	12 (12.00)	—
PI-based (yes versus no)	81 (63.78)	21 (77.78)	60 (60.00)	0.088
ART era				
Pre-ART (<1996)	10 (7.87)	1 (3.70)	9 (9.00)	0.151
Phase in ART (1996–2001)	17 (13.39)	1 (3.70)	16 (16.00)	
Highly effective ART (>2001)	100 (78.74)	25 (92.59)	75 (75.00)	
*Comorbidities*				
Hepatitis A	0 (0%)	—	—	
Hepatitis B	5 (3.94%)	1 (3.70)	4 (4.00)	1.000
Hepatitis C	9 (7.09%)	2 (7.41)	7 (7.00)	1.000
Psychiatric history	21 (16.54%)	8 (29.30)	13 (13.00)	0.040
STI history	19 (14.96%)	3 (11.11)	16 (16.00)	0.762
TB history	27 (21.26%)	7 (25.93)	20 (20.00)	0.597
Malaria history	22 (17.32%)	4 (14.81)	18 (18.00)	1.000
Diabetes	8 (6.30%)	2 (7.41)	6 (6.00)	0.678
Hypertension	4 (3.15%)	1 (3.70)	3 (3.00)	1.000

^1^Missing data were excluded from analysis. ^2^Column percentages.

**Table 3:** Bivariable and multivariable logistic regression to assess associations with adverse obstetrical outcomes using generalized estimating equations to control for year of birth (*n* = 127).

Variables (*m* = missing)	OR (95% CI)	*p* value	AOR (95% CI)	*p* value
*Obstetrical factors at time of birth*				
Parity				
1	Ref.			
2	0.74 (0.25–2.19)	0.580		
3+	1.18 (0.29–4.78)	0.821		
Gravidity				
1	Ref.			
2	1.40 (0.53–3.68)	0.500		
3+	1.40 (0.34–5.75)	0.644		
Mode of delivery (*m* = 11)				
Vaginal	Ref.	0.724		
Caesarian section	1.29 (0.73–2.29)			
*Demographic variables*				
Age at delivery				
<20 years	2.26 (1.00–5.09)	0.049	3.35 (1.04–1.46)	0.043
20–35 years	Ref.		Ref.	
36+ years	1.15 (0.40–3.27)	0.846	0.88 (0.26–3.03)	0.846
Ethnicity				
African migrant	1.01 (0.51–2.03)	0.891		
Other	Ref.			
Immigrant status				
Nonimmigrant	Ref.		Ref.	
<10 years	5.88 (1.26–27.36)	0.023	7.24 (1.30–40.28)	0.024
≥10 years	1.95 (0.21–18.56)	0.560	2.53 (0.19–33.29)	0.480
Health insurance				
Yes	Ref.	1.00		
No	0.75 (0.13–4.53)			
Living with partner (*m* = 27)				
Yes	Ref.	0.883		
No	1.02 (0.43–2.42)			
Housing (*m* = 11)				
Stable	Ref.	0.720		
Unstable	1.24 (0.35–4.40)			
Intravenous drug use				
Yes	1.59 (0.45–5.65)	0.476		
No	Ref.			
Smoking history^1^				
Yes	2.63 (1.14–6.04)	0.023		
No	Ref.			
*HIV related variables*				
Diagnosis time frame (*m* = 25)				
Preconception	Ref.	0.762		
During pregnancy/after delivery	1.23 (0.35–4.31)			
CD4 cell count (*m* = 21)				
<200	0.58 (0.04–9.38)	0.428		
200–499	0.15 (0.02–1.32)			
>500				
Virological suppression (*m* = 20)				
Yes	Ref.	0.229		
No	0.56 (0.22–1.46)			
ART regime				
Yes	Ref.			
No				
ART combination				
Any ART (yes versus no)	0.89 (0.27–3.00)	0.864		
NRTI (yes versus no)	1.13 (0.40–3.25)	0.814		
NRTI only (yes versus no)	0.26 (0.03–2.02)	0.198		
NNRTI-based (yes versus no)^1^	—	—		
PI-based (yes versus no)	0.44 (0.14–1.39)	0.164		
ART era^1^				
Pre-ART (<1996)	0.35 (0.03–3.65)	0.151		
Phase in ART (1996–2001)	0.18 (0.04–0.95)	0.043		
Highly effective ART (>2001)	Ref.			
*Comorbidities*				
Hepatitis A^1^	—			
Hepatitis B	1.09 (0.15–7.84)	1.00		
Hepatitis C	1.09 (0.25–4.68)	1.00		
Psychiatric illness	2.77 (1.22–6.33)	0.04	2.85 (1.23–6.61)	0.014
STI history	0.71 (0.26–1.93)	0.762		
TB history	1.19 (0.45–3.17)	0.597		
Malaria history	0.85 (0.35–2.08)	1.00		
Diabetes	1.08 (0.45–2.59)	0.678		
Hypertension	1.33 (0.22–8.24)	1.00		

^1^Insufficient cell size to run multivariable analyses.
